# Using Iron-Manganese Co-Oxide Filter Film to Remove Ammonium from Surface Water

**DOI:** 10.3390/ijerph14070807

**Published:** 2017-07-19

**Authors:** Ruifeng Zhang, Tinglin Huang, Gang Wen, Yongpan Chen, Xin Cao, Beibei Zhang

**Affiliations:** 1 Key Laboratory of Northwest Water Resource, Environment and Ecology, MOE, Xi’an University of Architecture and Technology, Xi’an 710055, China; ruifengzhangtry@163.com (R.Z.); chenyongpansir@163.com (Y.C.); 15191856946@163.com (B.Z.); 2 Shanxi Key Laboratory of Environmental Engineering, Xi’an University of Architecture and Technology, Xi’an 710055, China; 3 Institute of Water Resources and Hydro-Electric Engineering, Xi’an University of Technology, Xi’an 710055, China; caoxin@xaut.edu.cn

**Keywords:** iron-manganese co-oxide, ammonium removal, surface water, chemical catalytic oxidation

## Abstract

An iron-manganese co-oxide filter film (MeO_x_) has been proven to be a good catalyst for the chemical catalytic oxidation of ammonium in groundwater. Compared with groundwater, surface water is generally used more widely and has characteristics that make ammonium removal more difficult. In this study, MeO_x_ was used to remove ammonium from surface water. It indicated that the average ammonium removal efficiency of MeO_x_ was greater than 90%, even though the water quality changed dramatically and the water temperature was reduced to about 6–8 °C. Then, through inactivating microorganisms, it showed that the removal capability of MeO_x_ included both biological (accounted for about 41.05%) and chemical catalytic oxidation and chemical catalytic oxidation (accounted for about 58.95%). The investigation of the characterizations suggested that MeO_x_ was formed by abiotic ways and the main elements on the surface of MeO_x_ were distributed homogenously. The analysis of the catalytic oxidation process indicated that ammonia nitrogen may interact with MeO_x_ as both ammonia molecules and ammonium ions and the active species of O_2_ were possibly ^•^O and O_2_^−^.

## 1. Introduction

Ammonium is one of the primary pollutants in water sources. Controlling the ammonium level in drinking water has become a prominent public health issue [1]. Excessive ammonium in drinking water can cause nitrification in the water distribution system, leading to many problems including corrosion, aesthetic issues (taste and odor), pH decrease, and biological instability [2]. Ammonium can also negatively affect free chlorine or chloramine residuals, which can lead to insufficient microbial disinfection in distribution systems [3]. However, it is difficult to remove using traditional drinking water treatment processes [4].

The general methods to remove ammonium from drinking water include break-point chlorination and biological filtration [5,6]. For break-point chlorination, low concentrations of ammonium can cause significant increases in the required chlorine (the theoretical consumption of 7.6 mg of Cl_2_ per mg of NH_4_^+^-N) and an excessive amount of chlorine may lead to the formation of undesirable chlorinated by-products [7]. Biological treatment is a popular method to remove ammonium. However, many water treatment plants in China have limited space and investment available to implement these processes [8]. In addition, at low water temperatures, biological treatment systems may perform poorly [9,10].

In recent studies, it was reported that ammonium and manganese could be removed from groundwater effectively by a filter system using iron-manganese co-oxides filter film (MeO_x_) coated sand as filter media and this co-oxides filter film has been proven to be efficient for the chemical catalytic oxidation of ammonium [11]. Although the utilities of the naturally formed or synthetic manganese oxide coated filter media to remove manganese has been reported widely [12,13,14], only few research groups have studied the removal of ammonium and the oxidation of ammonium was generally considered to be done mainly by microorganisms [15]. However, this co-oxides filter film was proven to be a good catalyst for the chemical catalytic oxidation of ammonium. The MeO_x_ filter system may be an alternative process for ammonium removal in drinking water treatment. The MeO_x_ could form quickly, taking less than 30 days, by adding potassium permanganate to the groundwater to oxide the Fe^2+^ and Mn^2+^ and then filtrating with a quartz sand filter [11]. In comparison, for traditional biological processes, the natural biofilm formation may take 3–4 months (Rittmann 1984). The study also showed that ammonium concentrations in the effluent of the MeO_x_ filter system used for treating groundwater was below 0.2 mg/L when the influent ammonium concentration in the influent fluctuated in the range of 0.8–2.4 mg/L [11]. Therefore, it may be an alternative method for ammonium removal in drinking water treatment.

However, few corresponding investigations were conducted on surface water treatment. Compared with groundwater, surface water sources are used more widely and generally have some characteristics that make ammonium more difficult to remove, such as low water temperatures in winter, seasonal fluctuation in the water quality, low alkalinity, low pH, and complex pre-treatment processes [9,16,17]. Especially for the surface water without Fe^2+^ and Mn^2+^, the situation may be more complex for the application of MeO_x_. Therefore, it is meaningful and necessary to investigate the applicability of MeO_x_ for ammonium removal from surface water.

In this study, MeO_x_ that had been operated continuously for about four years for groundwater treatment was used for ammonium removal from a surface water source in northwest China. The experiments concentrated mainly on the following objectives: (1) the applicability of MeO_x_ for ammonium removal from surface water, (2) ammonium removal rates by MeO_x_ from surface water, and (3) the mechanism of ammonium removal by MeO_x_ in surface water.

## 2. Materials and Methods

### 2.1. Pilot-Scale Filter System

**Figure 1:**
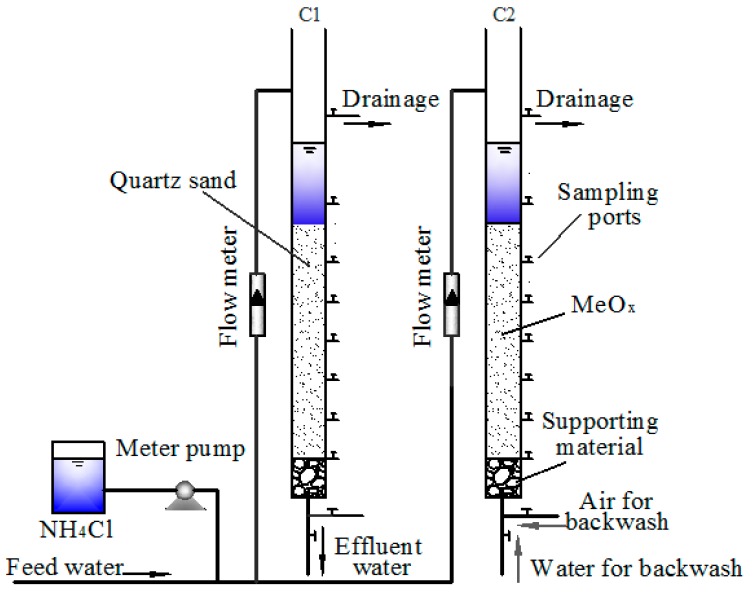
Schematic of a pilot-scale filter system.

The filter columns were made of two identical plexiglas tubes with an inner diameter of 100 mm and the active filter beds fixed in the filter columns were 120 cm (Figure 1). In filter column C1, the filter bed was virgin quartz sand for use as a blank control. In filter column C2, the active filter bed consisted of MeO_x_-coated sand formed in the continuously-operated groundwater filter system. The diameters of both the MeO_x_-coated sand and virgin quartz sand were 1–2 mm. The feed water for this filter system was the treated water coming from a sedimentation tank. First, NH_4_Cl (Kemiou Co., Ltd., Tianjin, China) stock solution was added to the feed water to adjust the ammonium concentration. Then, the synthesized water was treated by a MeO_x_ filter.

### 2.2. Effects of Ammonium Concentration and Filtration Rate

First, the applicability of MeO_x_ for surface water treatment was investigated at steady operational conditions. The average filtration rate and influent ammonium concentration were about 7 m/h and 2 mg/L, respectively. Then, the ammonium removal efficiency of the MeO_x_ was investigated at different influent ammonium concentrations (1.0, 1.4, 1.7, 2.1, 2.4, and 2.7 mg-NH_4_^+^-N/L at a filtration rate of 7 m/h) and different filtration rates (4, 7, 9, 11, and 13 m/h at an influent ammonium concentration of 2.0 mg-NH_4_^+^-N/L). Each operational condition lasted for two days, and two sets of parallel samples were collected for analysis.

The volumetric ammonium removal rates (VARR) of the filter sands were calculated as described by Lee et al. [16]. The corresponding equation is:

VARR = *Q*(*C_a,in_* – *C_a,out_*)/A *Δz* (1)

where *Q* is the volumetric flow rate (L/h), *C_a,in_* is the influent ammonium concentration (mg/L), *C_a,out_* is the effluent concentration (mg/L), A is the cross sectional area of the filter (m^2^), and *Δz* is the active depth of the filter bed (m, which was 40 cm in this experiment).

### 2.3. Effect of Temperature 

The effect of temperature on ammonium removal was studied by determining the specific ammonium removal rates (SARR) of the filter media at different water temperatures and by investigating the ammonium removal performance of the MeO_x_ filter at 6–8 °C (the lowest water temperature in winter). SARR of MeO_x_ was measured using the methods described by De Vet et al. [18], which was calculated from the linear trend of the mass of the removed ammonium as a function of time and expressed as mg-NH_4_^+^-N per Kg of MeO_x_-coated sand per hour (mg-NH_4_^+^-N/(kg h)).

### 2.4. Inactivation Method

In this study, ozone (O_3_) was used to inactivate bacteria on the surface of the MeO_x_ in order to distinguish between the biological and catalytic chemical oxidation for ammonium removal in the surface water. O_3_ was produced by an ozone generator (Guo LinCF-G-3-10 g, Qingdao Guo Lin Industry Co., Ltd., Qingdao, China). As the backwashing finished, tap water without ammonium was fed into the filter column for about 1 h. Then, the inlet valve of the filter was closed and the water level in the filter was controlled to keep the media submerged about 1 m. Finally, ozone gas was introduced to the MeO_x_-sand filter for about 6 h continuously. In this process, the concentration of O_3_ in the liquid was maintained at about 4.5–6.8 mg/L. The ammonium removal efficiency was determined before and after inactivation. The MeO_x_ was collected and immediately stored at 4 °C.

### 2.5. Characterization Methods

The sands were washed with deionized water several times. Then, they were frozen and vacuum-dried by a freeze dryer (FD-1D-50, Beijing Medical Kang Bo Experimental Instrument Co., Beijing, China) and kept in sealed vacuum tubes until analysis could be performed. The specific surface area and pore size of the filter sands were obtained using the BET method (Quantachrome-AUTOSORB-1C). The specific surface area of the MeO_x_-coated sand was as high as 24.06 m^−2^ g^−1^, while the corresponding value of normal quartz sands was only 0.102 m^−2^ g^−1^ (Table S1). The surface morphologies and elemental distributions on the filter sands were determined using a Scanning Electron Microscope (ZEISS-SUPRA55, Bruker, Karlsruhe, Germany) with an X-ray energy spectrum instrument (AztecX-Max80, Oxford Instruments, London, UK).

### 2.6. Analytical Methods

The concentrations of ammonium, nitrite, nitrate, and alkalinity were determined following the Chinese National Standard Methods [19]. pH and the concentration of dissolved oxygen (DO) were measured using a pH meter (PH-25, Leici Co., Ltd., Shanghai, China) and dissolved oxygen meter (JPB-607A, Leici Co., Shanghai, China), respectively. The O_3_ concentrations were determined using indigo spectrophotometry [20] (Bader and Hoigné 1981). The suspension of the bacteria was obtained by shaking (5 h, 150 r/min, SHZ-82A, Changzhou Guohua Electric Co., Ltd., Changzhou, China) and sonication (5 min, KQ-500DE, Kunshan Ultrasonic Instrument Co., Ltd., Kunshan, China) in sterile phosphate buffered solution [11,21]. The biomass of ammonium-oxidizing bacteria (AOB) and nitrite-oxidizing bacteria (NOB) present on the MeO_x_ was enumerated using the most probable number method according to the reported method [22,23].

## 3. Results and Discussion

### 3.1. The Applicability of MeO_x_ for Ammonium Removal from Surface Water

**Figure 2:**
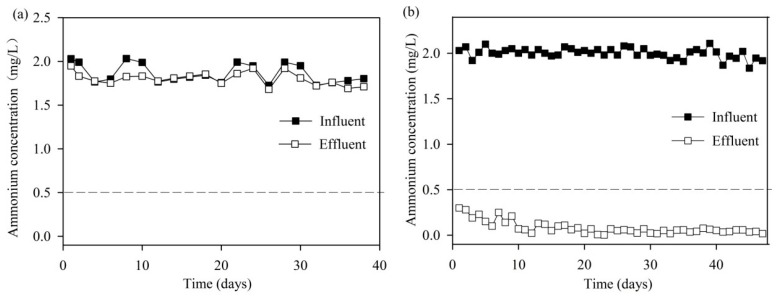
Ammonium removal performance of (**a**) normal quartz sands filter and (**b**) MeOx filter for treating surface water. Water temperature was 13–16 °C.

Figure 2a shows that the ammonium removal efficiency of the normal quartz sand filter was always lower than 10% during 40 days of operation. Figure 2b gives an overview of ammonium removal performance in surface water using the MeO_x_ filter for about 47 days for surface water treatment. In the first 10 days, the effluent ammonium concentration was a bit higher, but still below 0.5 mg/L. After operating for about 10 days, the average ammonium concentration in the effluent stabilized was lower than 0.1 mg-NH_4_^+^-N/L and the removal efficiency was higher than 90%. This performance was comparable with the ammonium removal from groundwater at 18–21 °C (Figure S1). It was demonstrated that the MeO_x_ filter system, established from the groundwater water treatment plant, is applicable for the almost immediate removal of ammonium from surface water, even though there were remarkable differences in water qualities between the surface water and groundwater (Table 1). However, an accommodation time of 3–4 weeks is required for the traditional biofilter system [24].

### 3.2. Effect of the Ammonium Concentration

**Figure 3:**
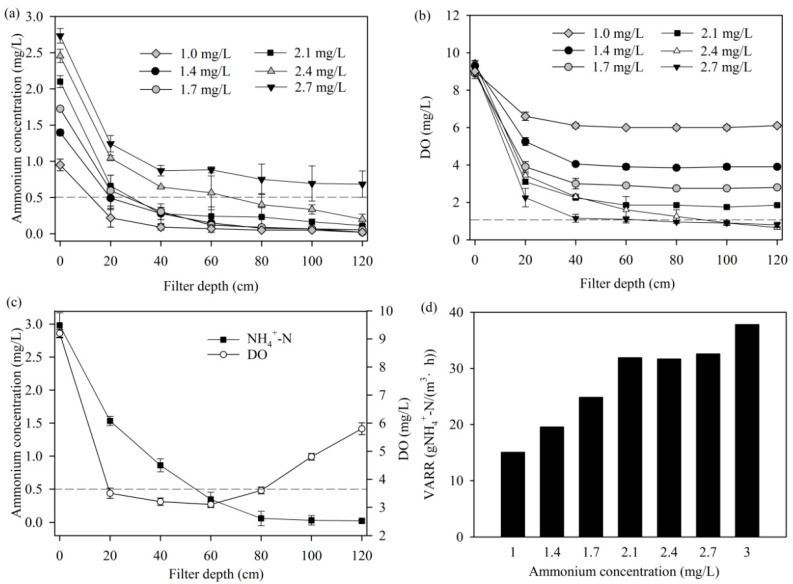
(**a**) Ammonium concentration depth profiles, (**b**) dissolved oxygen (DO) concentration depth profiles, (**c**) ammonium and DO concentration depth profiles with compressed air forcing into the filter from the bottom, and (**d**) the volumetric ammonium removal rates (VARR) in 0–40 cm filter depth.

Figure 3a shows the ammonium concentration depth profiles at different influent ammonium concentrations. It indicates that the ammonium was removed mainly at 0–40 cm filter depth. The maximum safety influent ammonium concentration was about 2.4 mg/L. However, the ammonium concentrations were almost unchanged in 40–120 cm filter depth, and the effluent ammonium concentration was as high as 0.7 mg/L when the influent ammonium concentration was 2.7 mg/L. The corresponding dissolved oxygen (DO) concentration depth profiles show that the DO concentration was decreased to about 1 mg/L at 40–120 cm filter depth (Figure 3b). It suggests that a shortage of dissolved oxygen at the bottom of the filter bed was possibly the limiting factor for ammonium removal. Similar results were also found for the groundwater, in which the effluent ammonium concentration was higher (influent 2.4 mg/L, effluent 1.0 mg/L) because of the lower DO concentrations (6.5–7.0 mg/L) [11]. The almost unchanged concentrations of DO at 40–120 cm filter depth also indicate that the dissolved oxygen was difficult to use at concentrations below 1 mg/L. Ammonium could be completely removed when compressed air was forced into the filter from the bottom layer, even for influent ammonium concentrations as high as 3.0 mg/L (Figure 3c).

Figure 3d shows that the volumetric ammonium removal rate (VARR) of MeO_x_ at 0–40 cm filter depth increased noticeably when the influent ammonium concentration was increased from 1.0 mg/L to 2.1 mg/L, and changed slightly when the influent ammonium concentration further increased up to 2.1–2.7 mg/L. The VARR of MeO_x_ at 0–40 cm filter depth increased again when compressed air was forced into the filter (at an ammonium concentration of 3.0 mg/L). It suggests that the ammonium removal efficiency of the MeO_x_ was affected by the ammonium concentration when the concentration was below 2.1 mg/L and was limited by DO concentration when the ammonium concentration was above 2.1 mg/L.

### 3.3. Effect of the Filtration Rates

**Figure 4:**
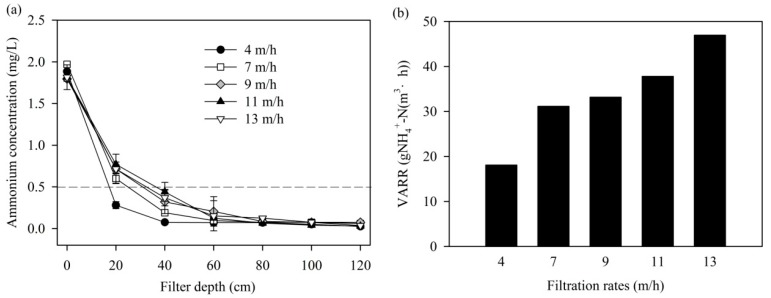
(**a**) Ammonium concentration depth profiles and (**b**) VARR at 0–40 cm filter depth at different filtration rates.

As shown in Figure 4a, the depth profiles of ammonium concentration increased slightly and effluent ammonium concentrations were lower than 0.2 mg/L when the filtration rates increased from 4 m/h to 13 m/h. The VARR of MeO_x_ at the 0–40 cm filter depth was increased by 1.59 times when the filtration rates were increased from 4 m/h to 13 m/h (Figure 4b). This suggests that ammonium removal from the MeO_x_ filter was probably mass-transfer limited [24].

### 3.4. Effect of Temperature

**Figure 5:**
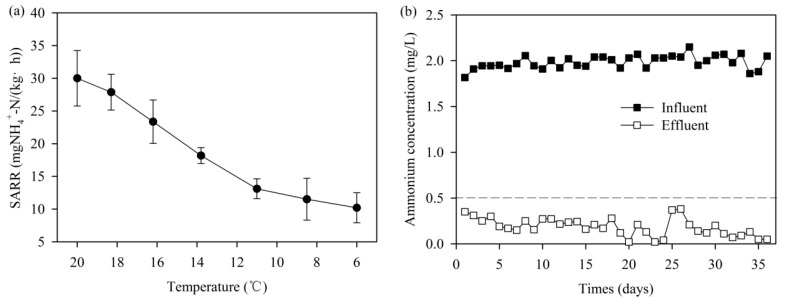
(**a**) Specific ammonium removal rates (SARR) of MeOx at different water temperatures in a lab-scale experiment and (**b**) ammonium removal performance of the MeOx filter in winter (6–8 °C).

The experimental results showed that the SARR would decrease with decreasing temperature (Figure 5a). In the temperature range of 13–16 °C, the average SARR was 18.18–23.26 mg-NH_4_^+^-N/(kg h) (mg-NH_4_^+^-N per Kg of MeO_x_-coated sand per hour) and it was decreased by 43.89–56.34% at 6 °C, which was the lowest surface water temperature at the study site. However, pilot-scale experimental results indicate that ammonium could still be reduced to less than 0.5 mg/L and the average ammonium removal efficiency was still up to 90.6% at the temperature of 6–8 °C (Figure 5b). A previous study proposed that only 10–40% of ammonium was removed at 4–10 °C in a pilot-scale biological filter [9]. It indicates that MeO_x_ could remove ammonium better than traditional biological processes at low water temperature.

### 3.5. Effect of Inactivation

Previous studies indicated that the damage to the structure of the MeO_x_ caused by ozone or hydrogen peroxide inactivation was negligible [11,25]. After being treated with ozone, the biomass of AOB and NOB on the MeO_x_ was decreased by 99.17% and 99.58% respectively (Table S2). Thus, the effect of biological oxidation on ammonium removal could be excluded.

**Figure 6:**
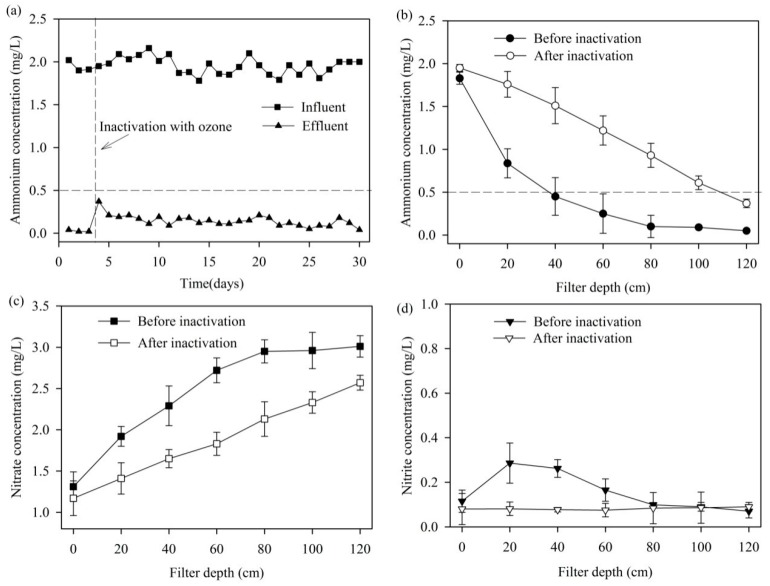
Ammonium removal performances of the MeOx filter before and after inactivation: (**a**) influent and effluent ammonium concentrations, (**b**) ammonium, (**c**) nitrate and (**d**) nitrite concentration depth profiles.

The average ammonium concentration in effluent was about 0.15 mg/L after inactivation (Figure 6a), although the filter depth required to remove ammonium to meet the permit limit expanded to the entire filter bed (Figure 6b). Therefore, ammonium could be removed effectively by the MeO_x_ only through the pathway of chemical catalytic oxidation.

For the MeO_x_ at 0–80 cm filter depth, the volumetric ammonium removal rates were decreased by 41.05% (calculated by Figure 6b) and the removed ammonium was almost all oxidized to nitrate (Figure 6c,d) after inactivation. It suggested that the removal capability of MeO_x_ included both biological (accounted for about 41.05%) and chemical catalytic oxidation (accounted for about 58.95%).

### 3.6. The Surface Morphology and Elemental Distribution of MeO_x_

**Figure 7:**
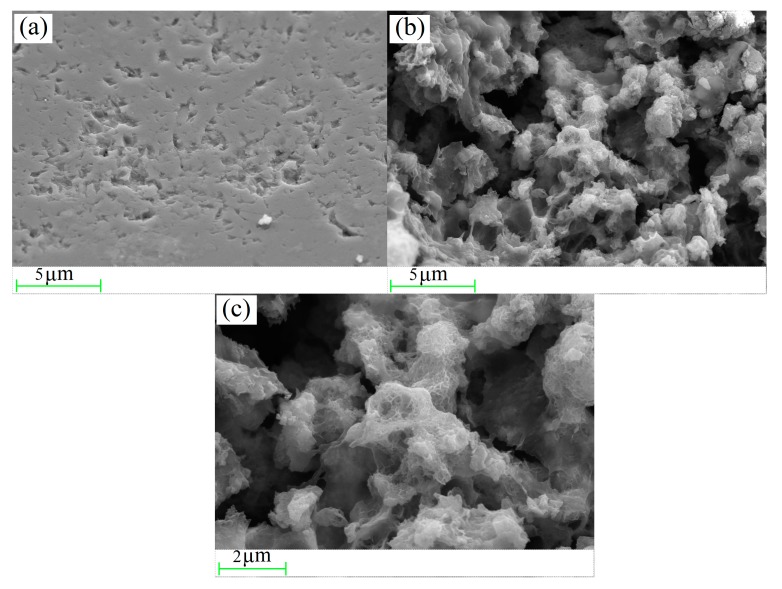
SEM images of (**a**) normal quartz sand magnified 5000 times, (**b**) MeOx magnified 5000 times, and (**c**) MeOx magnified 10,000 times.

SEM images for normal quartz sand and MeO_x_-coated sand are shown in Figure 7. In contrast to the flat surface of the normal quartz sand, the surface of MeO_x_ was rough and porous. The SEM micrographs also indicate that MeO_x_ had coral- or sponge-like structures, meaning that the formation of MeO_x_ follows a typical physicochemical procedure [26] and this structure may have strong autocatalytic oxidation capability for Mn^2+^ [27]. In this study, it may be favorable for the chemical catalytic oxidation of ammonium.

**Figure 8:**
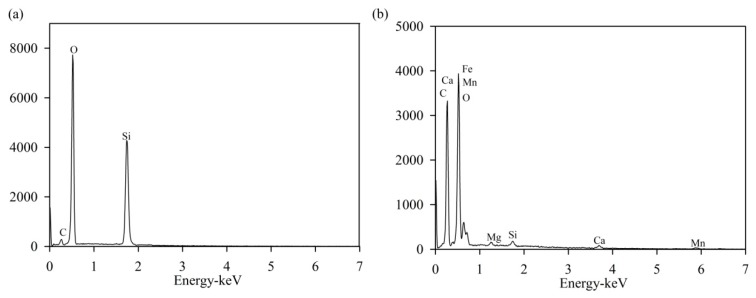
The EDS images of (**a**) normal quartz sand and (**b**) MeO_x_.

The energy dispersion spectra (EDS) were used to investigate the elemental composition of normal quartz sand and MeO_x_ (Figure 8). Primary elements on the surface of normal quartz sand were Si and O (Figure 8a), while those on the surface of MeO_x_ were C, O, Fe, Mn, Ca, Mg, and Si (Figure 8b). EDS mapping images (Figure S2) further indicate that these elements were homogeneously distributed on the surface of MeO_x_, which is different from the surface coatings observed on biofilter media [12]. These differences were possibly responsible for the high ammonium removal efficiency of MeO_x_.

### 3.7. Discussion on the Mechanism for Ammonium Oxidation

It has been widely demonstrated that ammonium is typically nitrified through a two-step process by different species of bacteria and archaea in a biological process for drinking water treatment [28]. In this study, the removal capability of MeO_x_ included both biological and chemical catalytic oxidation. This discussion mainly concentrated on the chemical catalytic oxidation of ammonium by MeO_x_ in surface water treatment.

The chemical catalytic oxidation of ammonium by MeO_x_ has been proven in groundwater [11,29]. The study of Guo et al. indicated that microorganisms may play a negligible role in the process of ammonium removal by MeO_x_ in groundwater [11]. Different from this opinion, the experimental results of the present study suggest that biological nitrification was also important in the MeO_x_ filter system in surface water treatment, although the chemical catalytic oxidation contributed about 58.95% of total ammonium removal. These differences may be caused by the different operating conditions and the different evaluation criteria in the treatment of surface water and groundwater (Table S3). The operating conditions in surface water treatment made the ammonium removal more difficult. However, the experimental results showed that ammonium could still be removed effectively by the MeO_x_ only through the pathway of chemical catalytic oxidation.

For the reaction mechanism of chemical catalytic oxidation, a pathway with five main steps was proposed in a previous study [11]. In this hypothesis, ammonium ions were assumed to be directly adsorbed onto the surface of the MeO_x_ before being oxidized, because the MeO_x_ was negatively charged. The dissolved oxygen was also considered to be adsorbed onto the surface of the MeO_x_, first to form the active state intermediate ^•^O that then reacted with the absorbed NH_4_^+^ to produce NH and H^+^.

However, other reaction pathways could also be proposed. The adsorption of ammonia nitrogen on the catalyst is divided into two categories, according to the type of acid sites: in one, the ammonia is adsorbed on Brønsted acid sites, labeled as NH_4_^+^, while in the other, the ammonia is adsorbed on Lewis acid sites, labeled as NH_3_ [30]. Both acid sites exist on the surface of the manganese oxide catalyst, and the surface is usually dominated by Lewis acid sites [31]. Consequently, the ammonium ion could also lose a proton to become ammonia and then be activated by the catalyst and be oxidized. O_2_^−^ may also be one type of active state intermediate of the dissolved oxygen on the co-oxide film [32]. Thermodynamic calculations indicate that the one-electron transfer reaction between the NH_4_^+^ and O_2_^−^ is favorable [33], suggesting that the O_2_ can also be transformed to O_2_^−^, which then reacts with ammonium.

## 4. Conclusions

This study has reported that a MeO_x_ filter system can remove ammonium from surface water at a removal efficiency comparable to that for groundwater. Specifically, the following conclusions can be drawn:

- The average ammonium removal efficiency of MeO_x_ was greater than 90%, even though the water quality changed dramatically and the water temperature was reduced to about 6–8 °C.
- The ammonium removal capability of MeO_x_ included both biological and chemical catalytic oxidation and chemical catalytic oxidation accounted for about 58.95%.
- MeO_x_ was possibly formed by abiotic ways and the main elements were homogenously distributed on the surface of MeO_x_.
- The analysis of the catalytic oxidation process indicated that ammonia nitrogen may interact with MeO_x_ as both ammonia molecules and ammonium ions and the active types of O_2_ were possibly O and O_2_^−^.

## Figures and Tables

**Table 1:** Water quality of surface water and groundwater used in the pilot-scale filter system.

Parameters	Unit	Surface Water	Ground Water
pH (before filtration)	–	7.7–7.9	8.0–8.2
pH (after filtration)	–	6.9–7.2	8.0–8.2
Temperature	°C	6.5–23.8	13.2–24.5
Alkalinity as (CaCO_3_)	mg/L	47–60	200–255
Dissolved oxygen	mg/L	9–10.0	1.35–3.5
Manganese	mg/L	<0.05	0.90–1.12
Total iron	mg/L	<0.10	0.85–1.19
Total phosphorus	mg/L	0.025–0.035	0.024–0.061

## Supplementary Materials

The following are available online at www.mdpi.com1660-4601/14/7/807/s1, Figure S1: Ammonium removal performance of MeOx filter for treating groundwater. Water temperature was 18–21 °C, Figure S2: SEM and EDS mapping images of MeO_x_; Table S1: Specific surface area, pore properties of the MeOx coated sand and normal quartz sand, Table S2: Biomass of nitrifying bacterial on MeOx before inactivation and after inactivation; Table S3: Comparison of the operating condition and evaluation criteria of the inactivation experiment in the study of Guo et al. [11] and current study.
