# Supplementary material for: Using Iron-Manganese Co-Oxide Filter Film to Remove Ammonium from Surface Water
Source: Int J Environ Res Public Health. 2017 Jul 19;14(7):807. doi: 10.3390/ijerph14070807 (PMC5551245; doi:10.3390/ijerph14070807)
Supplement: Supplementary file 1 [file ijerph-14-00807-s001.pdf]

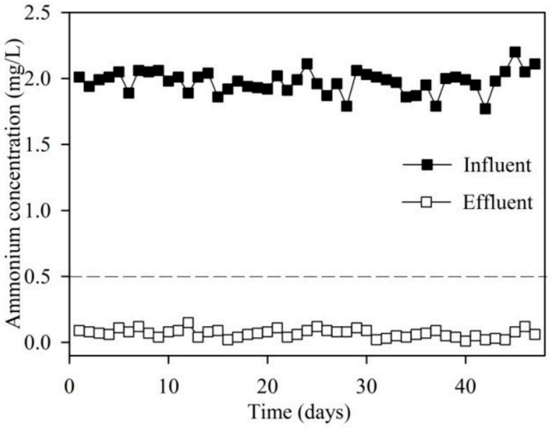

Figure S1. Ammonium removal performance of MeOx filter for treating groundwater. Water temperature was 18–21 °C.

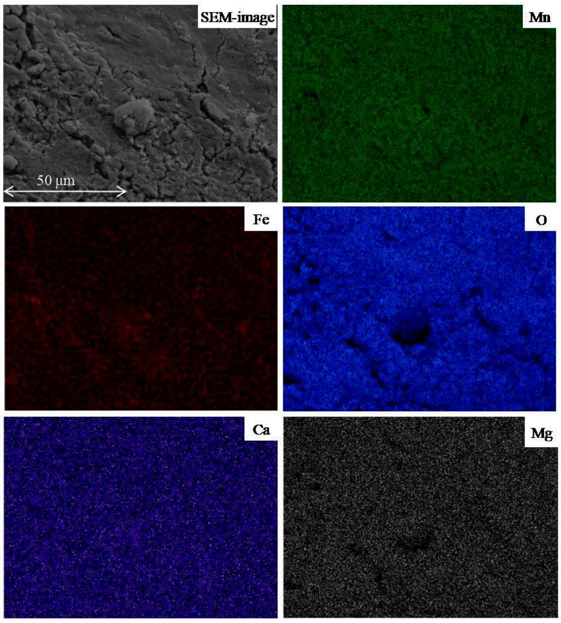

Figure. S2 SEM and EDS mapping images of MeO_x.

Table S1. Specific surface area, pore properties of the MeO_x coated sand and normal quartz sand.

Samples	Specific Surface Area (m^2/g)	Average Pore Diameter (nm)	Pore Volume (cm^3/g)
Normal quartz sand	0.102	11.23	0.000371
MeO_x coated sand	24.06	24.23	0.145693

Table S2. Biomass of nitrifying bacterial on MeO_x before inactivation and after inactivation.

Bacterial Species	MPN Numbers (cells/g MeO_x-coated sand)		Inactivation Rate
	before Inactivation	after Inactivation	
Ammonium-oxidizing bacteria	2.4×10^4	200	99.17%
Nitrite-oxidizing bacteria	3.8×10^4	160	99.58%

Table S3 Comparison of the operating condition and evaluation criteria of the inactivation experiment in the study of Guo *et al*. [11] and current study.

Reference	Type of Water	MeO_x	Water Temperature (°C)	Influent Ammonium (mg/L)	Evaluation Criterion
Guo *et al.* [11]	Groundwater	Operating for about one year	18.0	1.5	Ammonium removal rates of the whole filter system
This study	Surface water	Operating for about four years	8-10	2.0	VARR of the 0-80 cm filter depth
